# 97 Animals Are People Too: Comparing Human and Animal Burns in a Large Wildfire

**DOI:** 10.1093/jbcr/irae036.096

**Published:** 2024-04-17

**Authors:** Larissa Epstein, Jamie L Peyton, Alexandra Coward, Jason Heard, Soman Sen, Kathleen S Romanowski, Tina L Palmieri

**Affiliations:** UC Davis, Menlo Park, CA; University of California, Davis, California; UC Davis, Sacramento, CA; University of California - Davis Hosp, Sacramento, CA; UC Davis and Shriners Children's Northern California, Sacramento, CA; UC Davis, Menlo Park, CA; University of California, Davis, California; UC Davis, Sacramento, CA; University of California - Davis Hosp, Sacramento, CA; UC Davis and Shriners Children's Northern California, Sacramento, CA; UC Davis, Menlo Park, CA; University of California, Davis, California; UC Davis, Sacramento, CA; University of California - Davis Hosp, Sacramento, CA; UC Davis and Shriners Children's Northern California, Sacramento, CA; UC Davis, Menlo Park, CA; University of California, Davis, California; UC Davis, Sacramento, CA; University of California - Davis Hosp, Sacramento, CA; UC Davis and Shriners Children's Northern California, Sacramento, CA; UC Davis, Menlo Park, CA; University of California, Davis, California; UC Davis, Sacramento, CA; University of California - Davis Hosp, Sacramento, CA; UC Davis and Shriners Children's Northern California, Sacramento, CA; UC Davis, Menlo Park, CA; University of California, Davis, California; UC Davis, Sacramento, CA; University of California - Davis Hosp, Sacramento, CA; UC Davis and Shriners Children's Northern California, Sacramento, CA; UC Davis, Menlo Park, CA; University of California, Davis, California; UC Davis, Sacramento, CA; University of California - Davis Hosp, Sacramento, CA; UC Davis and Shriners Children's Northern California, Sacramento, CA

## Abstract

**Introduction:**

Although human burns have been studied in depth, there are few studies involving animal burns, and even fewer studies that compare the two. However, recent wildfires across the globe, like the 2019 Australian bushfire, have drawn attention to the plight of animals. Our study aims to compare effects of wildfire burns between humans and animals. Animals have adaptations that may protect them from burns: fur/feathers, agility, innate fight or flight response, and burrowing/swimming/flying abilities. We hypothesize that humans would have deeper burns and a higher mortality rate than their animal counterparts.

**Methods:**

After IRB approval, a retrospective chart review was conducted using electronic medical records for humans with burns in 2018 wildfires at a university hospital and burned animals treated at the veterinary hospital. Animals were split into 2 groups: companion animals (CA) and non-companion animals (NCA). Data collected included demographics, burn and hospitalization characteristics, treatment, discharge, and outcomes. Analysis was conducted with SAS statistical software, version 9.4 (SAS Institute, Cary, NC, USA) using Chi-squared and Kruskal-Wallis tests.

**Results:**

In all, 123 animals and 10 humans were analyzed. Ninety-four animals were identified as CA (dogs, cats) and 29 NCA (turtles, cattle, llamas, etc). All groups had mean total body surface area (TBSA) below the 20% TBSA resuscitation guidelines (70% human vs. 98.9% CA vs.72.5% NCA). There was no difference between location or burn severity, between groups. The most common location of burns in humans was the head (80%) and hands and feet (80%). Similarly, animals most commonly burned their paws (97.9% CA vs. 62.1% NCA). The median time to presentation was significantly different between humans, CAs, and NCAs (Day 0, 5, 8; p< 0.001). Humans and CAs sustained mainly 2nd degree burns (90% vs. 76.6%) while NCAs suffered mostly from 3rd degree burns (65.5%). Median number of hospital days (HD) between humans, CAs, and NCAs differed significantly between groups (16.5, 27, 8; p=0.025). Survival to discharge was highest in humans (90.0% human vs. 92.4% CA vs. 72.4% NCA, p=0.039). Humans were treated with surgery (100%). Fewer animals underwent surgical excision at initial presentation (16% CA vs.40.7% NCA p< 0.001).

**Conclusions:**

Humans and animals burned by wildfires had different injury extent, survival, and burn distribution. CAs had the most HDs, yet had the fewest surgeries. There are major differences in treatment between human and animal burn treatment and outcomes. Current animal burn care does not follow human medical standards.

**Applicability of Research to Practice:**

Most burn outcomes and research are focused on humans. More attention is being brought towards burned animals, which can be used to further research into animal care.

